# TMAO Activates the NLRP3 Inflammasome, Disrupts Gut–Kidney Interaction, and Promotes Intestinal Inflammation

**DOI:** 10.3390/ijms26157441

**Published:** 2025-08-01

**Authors:** Leyao Fang, Junxi Shen, Nenqun Xiao, Zhoujin Tan

**Affiliations:** 1 School of Traditional Chinese Medicine, Hunan University of Chinese Medicine, Changsha 410208, China; 18229704061@163.com (L.F.); shenjunxi816@163.com (J.S.); 2 Hunan Key Laboratory of Traditional Chinese Medicine Prescription and Syndromes Translational Medicine, Changsha 410208, China; 003793@hnucm.edu.cn; 3 School of Pharmacy, Hunan University of Chinese Medicine, Changsha 410208, China

**Keywords:** gut microbiota, trimethylamine N-oxide, gut–kidney axis, inflammasome, renal-associated diarrhea

## Abstract

Gut microbiota-derived trimethylamine N-oxide (TMAO) has been implicated in both intestinal and renal diseases; however, its specific role in modulating gut–kidney interactions remains unclear. This study aimed to investigate the effects of TMAO on gut–kidney crosstalk using a mouse model of diarrhea. Mice were divided into four groups: normal, model, TMAO, and TMAO + model. The normal group received sterile water, while the other groups were administered adenine + *Folium sennae*, TMAO, or a combination of TMAO and adenine + *Folium sennae*. Samples were collected to assess morphological changes in the colon and kidney, evaluate the colonic mucosal barrier and renal function, and measure NLRP3 inflammasome activity and inflammatory cytokine levels in colonic and renal tissues. TMAO levels and the gut microbiota composition were analyzed using 16S rRNA sequencing. The model group exhibited altered stool morphology, which was further aggravated by TMAO intervention. Both the model and TMAO + model groups exhibited significant damage to intestinal and renal tissues, along with compromised intestinal mucosal barriers and impaired renal function compared to controls. Inflammatory markers were elevated in these groups, with the TMAO + model group showing the most pronounced increases. Correlation analysis indicated significant relationships among TMAO levels, inflammasome activation, and inflammatory cytokines. The genera *Mucispirillum* and *Anaerotruncus* negatively correlated with TMAO, whereas *Parabacteroides* and *Parasutterella* genera positively correlated with TMAO. In conclusion, TMAO plays a critical role in modulating gut–kidney crosstalk by promoting inflammation, disrupting mucosal and renal integrity, and altering the gut microbial ecosystem.

## 1. Introduction

The gut microbiota plays a pivotal role in host metabolic and immune homeostasis, not only regulating intestinal health but also exerting systemic effects through the gut–organ axes. Among these, the gut–kidney axis has gained increasing attention as a bidirectional communication network in which microbiota-derived metabolites serve as key mediators. Metabolites of the gut microbiota can influence intestinal immunity, inflammatory responses, and mucosal barrier function, thereby contributing to the development and progression of intestinal disease [1]. Emerging evidence suggests that these microbial metabolites not only affect the intestine but also have systemic effects, including on the kidneys [2]. The “gut–kidney axis” theory highlights the bidirectional relationship between the intestines and kidneys, where gut microbiota metabolites act as crucial mediators of this interaction [3]. The kidneys and intestines play parallel roles in maintaining fluid metabolic equilibrium and eliminating metabolic waste [4]. In addition to their physiological collaboration, these two organs are also closely linked in disease pathology. Chronic kidney disease (CKD) is often accompanied by gastrointestinal symptoms such as diarrhea and vomiting [5]. The accumulation of metabolic waste products, including gut-derived uremic toxins, in CKD patients can trigger intestinal damage through mechanisms such as inflammation, oxidative stress, and immune responses [6]. Post-transplant diarrhea is also a common complication following kidney transplantation [7,8]. The “gut–kidney axis” theory suggests that gut microbiota and its metabolites can influence kidney function through various pathways, while kidney damage can also alter the gut microenvironment, creating a vicious cycle of “gut–kidney interaction.”

Among gut microbiota-derived metabolites, trimethylamine N-oxide (TMAO) has gained considerable attention due to its involvement in various disease processes. Extensive research has shown that TMAO is a detrimental metabolite produced by the metabolism of gut bacteria [9,10]. Dietary components like choline and L-carnitine are metabolized by intestinal microbial trimethylamine lyases into trimethylamine (TMA), and then oxidized by hepatic flavin-containing monooxygenase 3 (FMO3) to form TMAO, primarily excreted via the kidneys [11,12]. TMAO has detrimental effects on both intestinal and renal tissues. TMAO has been shown to decrease the expression of tight junction proteins, including occludin and zonula occludens-1 (ZO-1), while significantly raising markers of intestinal barrier disruption, such as diamine oxidase (DAO), leading to increased intestinal permeability [13]. Upon entering the bloodstream, TMAO induces systemic inflammatory responses, compromises the intestinal mucosal barrier, and exacerbates gut microbial dysbiosis, hence worsening diarrhea. Increased concentrations of TMAO in the bloodstream correlate with elevated levels of creatinine (Cr) and blood urea nitrogen (BUN), which exacerbate renal impairment and elevate mortality risk in patients [14]. TMAO accelerates the development of nucleotide-binding oligomerization domain-like receptor protein 3 (NLRP3) inflammasomes in renal tissues and activates cleaved interleukin-1 beta (IL-1β) and caspase-1 [15]. The NLRP3 inflammasome transforms pro-caspase-1 into Caspase1-p20, which then activates IL-1β and interleukin-18 (IL-18). In vitro studies have shown that TMAO can induce ferroptosis and apoptosis in renal tubular epithelial cells, contributing to renal injury [16,17]. Limited evidence also suggests that TMA may disrupt mitochondrial homeostasis and promote inflammation in intestinal epithelial cells [18]. Our previous research demonstrated that a mouse model of diarrhea exhibits concurrent intestinal and renal impairment, accompanied by gut microbiota dysbiosis and elevated TMAO levels [19,20]. Further investigation into the role of TMAO in gut–kidney axis regulation may provide key insights into the pathogenesis of related disorders.

This study seeks to investigate the effect of TMAO on gut–kidney interaction by assessing TMAO levels, intestinal barrier integrity, renal function, inflammasomes, and inflammatory cytokine profiles. Pathological analysis of intestinal and renal tissues, along with high-throughput sequencing of cecal contents, was conducted to elucidate how TMAO mediates gut–kidney axis disruption, with the exacerbation of diarrhea symptoms serving as a manifestation of this disruption.

## 2. Results

### 2.1. General Conditions of Mice

**Figure 1:**
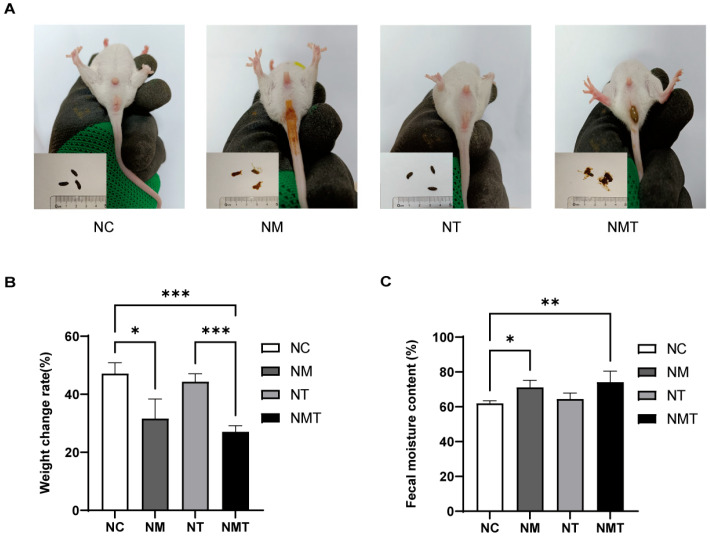
General condition of mice in each group. (**A**) Fecal morphology and perianal condition. (**B**) Body weight change rate. (**C**) Fecal moisture content. Data are presented as mean ± SD. * *p* < 0.05, ** *p* < 0.01, *** *p* < 0.001. NC: normal group, NM: model group, NT: TMAO group, NMT: TMAO + model group.

Mice in the normal group exhibited favorable mental states, rapid responses, normal feces, and clean perianal areas. Mice in the TMAO group demonstrated normal mental states and rapid responses. Mice in the model and TMAO + model groups, on the other hand, showed sluggish responses, reduced spontaneous activity, loose and unformed feces, and poor perianal cleanliness, with some exhibiting loose stool adhesion around the anus (Figure 1A). The model group gained less weight than the normal group (*p* < 0.05), and the TMAO + model group had a considerably lower body weight change rate than both the normal and TMAO groups (*p* < 0.001; Figure 1B). Figure 1C shows that the model group had higher fecal water content than the normal group (*p* < 0.05), whereas the TMAO + model group had considerably lower fecal water content (*p* < 0.01).

### 2.2. Effect of TMAO on Renal Histology and Function in Mice

**Figure 2:**
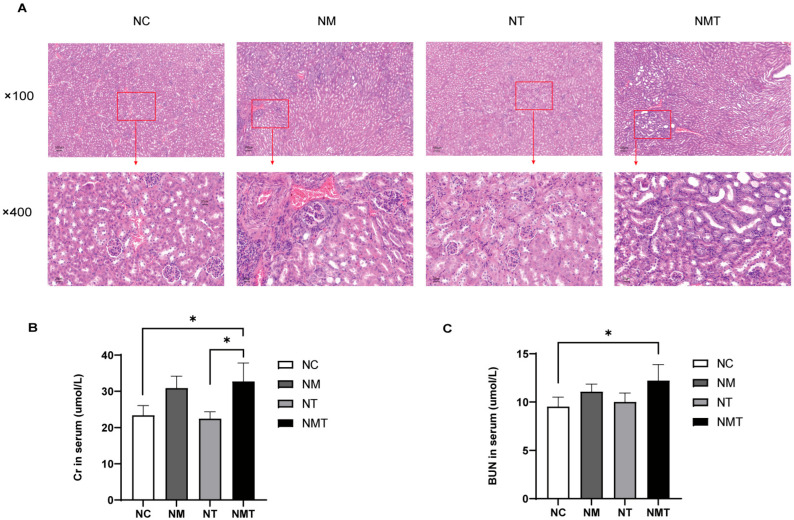
Renal histology and function in mice. (**A**) Representative kidney tissue sections at different magnifications (×100, ×400). Scale bars: 100 µm, 20 µm. (**B**) Serum Cr levels. (**C**) Serum BUN levels. The data are presented as mean ± SD. * *p* < 0.05. NC: normal group, NM: model group, NT: TMAO group, NMT: TMAO + model group.

Figure 2A shows the pathological section results of renal tissues. In the normal group, the glomeruli had normal volume, and the capsular space was clear. Renal tubules showed no atrophy of the tubular walls or lumen dilation, and the interstitium lacked significant inflammatory cell infiltration. The model group showed mesangial proliferation, increased glomerular volume, and poorly defined the capsular space. Renal tubules showed varied degrees of dilatation, including larger lumens and degenerative edematous tubular walls. Renal interstitial edema, congestion, and inflammatory cell aggregation were seen. The TMAO + model group exhibited structural damage to the renal cortex, characterized by irregularly shaped and enlarged glomeruli, as well as poorly defined gaps in the capsular space. Tubular structures displayed irregular lumens and congestion, accompanied by a significant increase in inflammatory cells within the observed area. Interstitial regions exhibited infiltration of inflammatory cells alongside dilated and congested blood vessels. Cr levels were significantly higher in both the model group and the TMAO + model group compared to the normal group (*p* < 0.05) (Figure 2B). Serum BUN levels were significantly elevated in the TMAO + model group (*p* < 0.05) (Figure 2C), whereas the increase in BUN levels in the model group was not statistically significant (*p* > 0.05). The TMAO group exhibited no significant differences in Cr or BUN levels when compared to the normal group (*p* > 0.05).

### 2.3. Effects of TMAO on Colonic Tissue and Intestinal Barrier Function in Mice

**Figure 3:**
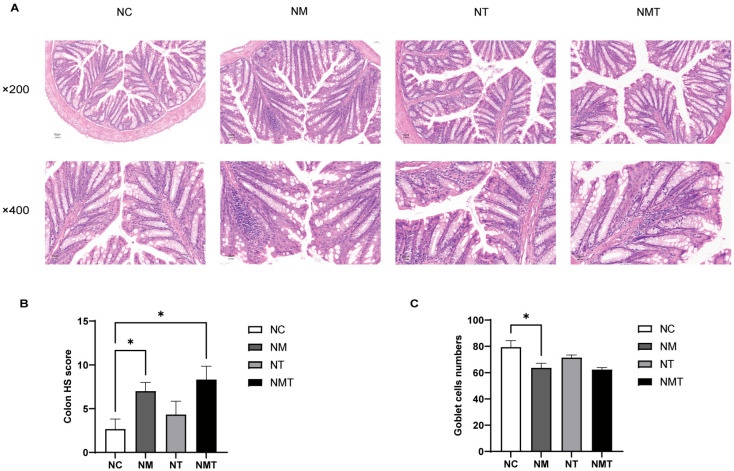
Colonic histology in mice of each group. (**A**) Representative images of colonic sections at different magnifications (×200, ×400). Scale bars: 50 µm, 20 µm. (**B**) HS scores of colonic tissue. (**C**) Goblet cell counts. Data are expressed as mean ± SD. * *p* < 0.05. NC: normal group, NM: model group, NT: TMAO group, NMT: TMAO + model group.

The intestinal tissue from the normal group exhibited intact crypt and mucosal structures (Figure 3A). In the model group, colonic tissue sections showed inflammatory cell infiltration, disrupted glandular structures, and a decrease in goblet cell numbers. Compared to the model group, the colonic tissue damage in the TMAO + model group was more severe, with wider and more extensive inflammatory cell infiltration and fewer goblet cells. The TMAO group displayed relatively intact crypt and mucosal structures, with limited inflammatory cell presence. The colonic sections were evaluated using the histopathological scoring (HS) criteria [21]. Pathological scores were markedly elevated in the model and TMAO + model groups relative to the normal group (*p* < 0.05), whereas the TMAO group exhibited a minor increase that lacked statistical significance (*p* > 0.05) (Figure 3B). Following H&E staining, goblet cells exhibited vacuolation, and Fiji (Image J 2.14.0/1.54f) facilitated the enumeration of all goblet cells within a single intact crypt structure [22]. Ten crypt structures were analyzed for each section. In comparison to the normal group, the model group demonstrated a significant decrease in goblet cell numbers (*p* < 0.05). Conversely, the TMAO and TMAO + model groups presented a lower count of goblet cells relative to the normal group, albeit without statistical significance (*p* > 0.05) (Figure 3C).

**Figure 4:**
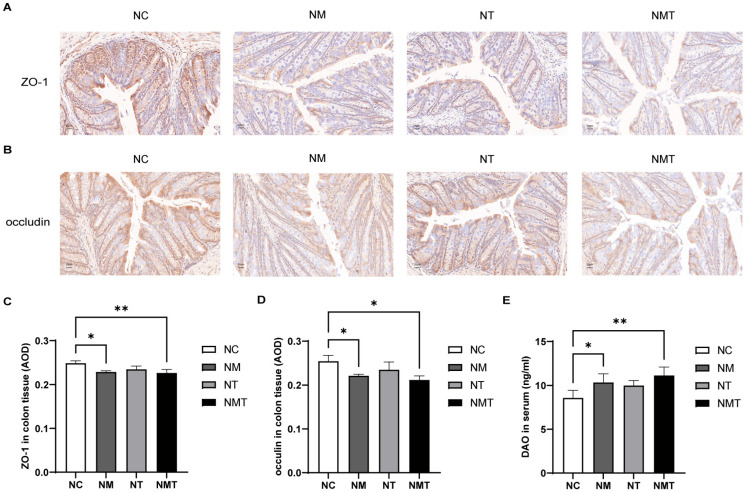
Intestinal mucosal barrier-associated molecules in different groups of mice. (**A**) ZO-1 protein expression in colonic tissue (IHC, ×400). Scale bar: 20 µm. (**B**) Occludin protein expression in colonic tissue (IHC, ×400). Scale bar: 20 µm. (**C**) Average optical density of ZO-1 protein in colonic tissue (*n* = 3). (**D**) Average optical density of occludin protein in colonic tissue (*n* = 3). (**E**) Serum DAO levels. Data are expressed as mean ± SD. * *p* < 0.05, ** *p* < 0.01. NC: normal group, NM: model group, NT: TMAO group, NMT: TMAO + model group.

In comparison to the normal group, ZO-1 protein expression in colonic tissue was diminished in the model group (*p* < 0.05) and markedly reduced in the TMAO + model group (*p* < 0.01) (Figure 4A,C). Occludin protein levels in colonic tissue were similarly reduced in both the model group and the TMAO + model group (*p* < 0.05) (Figure 4B,D). As shown in Figure 4E, serum DAO levels were significantly reduced in both the model group and the TMAO + model group when compared to the normal group (*p* < 0.05 and *p* < 0.01, respectively).

### 2.4. Colonic Inflammasomes and Inflammatory Cytokines

**Figure 5:**
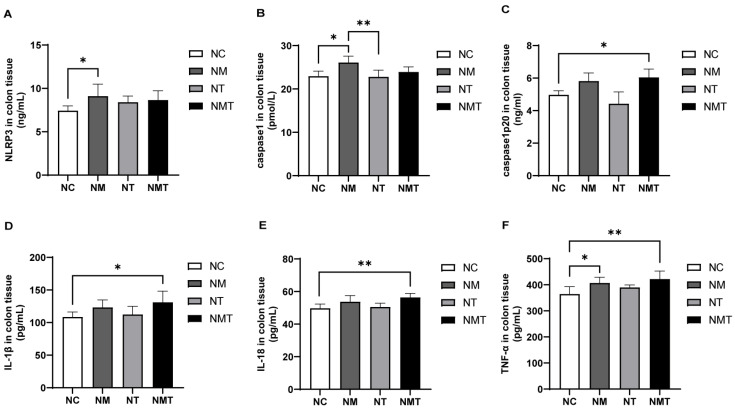
Changes in inflammasome-associated molecules and inflammatory cytokines in colonic tissues of mice. (**A**) NLRP3 levels in colonic tissues. (**B**) Caspase-1 levels in colonic tissues. (**C**) Caspase1-p20 levels in colonic tissues. (**D**) IL-1β levels in colonic tissues. (**E**) IL-18 levels in colonic tissues. (**F**) TNF-α levels in colonic tissues. Data are presented as mean ± SD. * *p* < 0.05; ** *p* < 0.01. NC: normal group, NM: model group, NT: TMAO group, NMT: TMAO + model group.

In comparison to the normal group, the model group exhibited significantly elevated levels of NLRP3 and caspase-1 in colonic tissue (*p* < 0.05). In the TMAO + model group, NLRP3 and caspase-1 levels appeared higher than in the normal group, but the differences were not statistically significant (*p* > 0.05). Caspase1-p20 levels were significantly elevated in the colonic tissue of the TMAO + model group (*p* < 0.05), while the model and TMAO groups exhibited numerical increases that did not reach statistical significance (*p* > 0.05). IL-1β levels were significantly higher in the colonic tissue of the TMAO + model group relative to the normal group (*p* < 0.05), whereas the model group showed a non-significant upward trend (*p* > 0.05). The levels of IL-18 and TNF-α in the colonic tissue of the TMAO + model group were significantly increased (*p* < 0.01). TNF-α levels in the model group were also higher than those in the normal group (*p* < 0.05), while the increase in IL-18 levels did not reach statistical significance (*p* > 0.05). The TMAO group did not show significant changes in inflammatory cytokine levels compared to the normal group. Additionally, when compared with the model group, IL-1β, IL-18, and TNF-α levels in the TMAO + model group were numerically higher, but the differences were not statistically significant (*p* > 0.05) (Figure 5).

### 2.5. Kidney Inflammasomes and Inflammatory Cytokines

**Figure 6:**
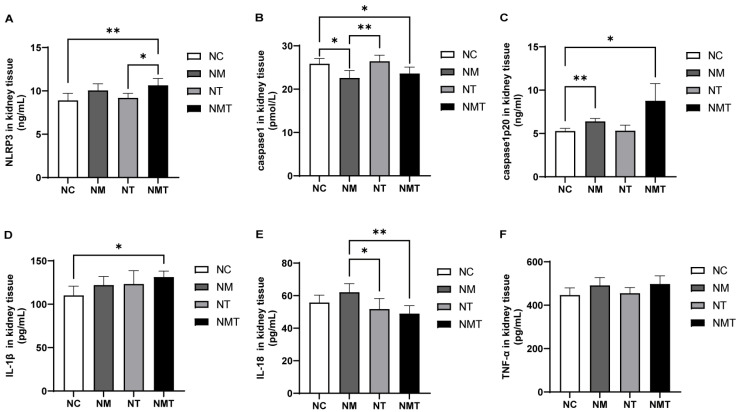
Changes of inflammasome-associated molecules and inflammatory cytokines in kidney tissues across groups. (**A**) NLRP3 levels in kidney tissue. (**B**) Caspase-1 levels in kidney tissue. (**C**) Caspase-1-p20 levels in kidney tissue. (**D**) IL-1β levels in kidney tissue. (**E**) IL-18 levels in kidney tissue. (**F**) TNF-α levels in kidney tissue. Data are presented as mean ± SD. * *p* < 0.05; ** *p* < 0.01. NC: normal group, NM: model group, NT: TMAO group, NMT: TMAO + model group.

Compared with the normal group, the TMAO + model group exhibited a significant increase in NLRP3 levels in kidney tissue (*p* < 0.01), whereas the model group exhibited a non-significant upward trend (*p* > 0.05) (Figure 6). Levels of Caspase1-p20 were elevated in both the model and TMAO + model groups (*p* < 0.01, *p* < 0.05), whereas Caspase-1 levels decreased in these groups (*p* < 0.05). The TMAO group exhibited no significant alterations in inflammasome-related markers within kidney tissues when compared to the normal group. Although inflammasome-related markers in the TMAO + model group appeared higher than those in the model group, the differences were not statistically significant (*p* > 0.05). In comparison to the normal group, IL-1β levels in the kidney tissues of the TMAO + model group were significantly elevated (*p* < 0.05), whereas TNF-α levels showed a non-significant upward trend (*p* > 0.05). IL-18 levels in the TMAO + model group were significantly lower than in the model group (*p* < 0.01). In the model group, IL-1β, IL-18, and TNF-α levels were numerically higher than those in the normal group, but none of these differences reached statistical significance (*p* > 0.05).

### 2.6. TMAO and TMA Levels Are Altered in Mice

**Figure 7:**
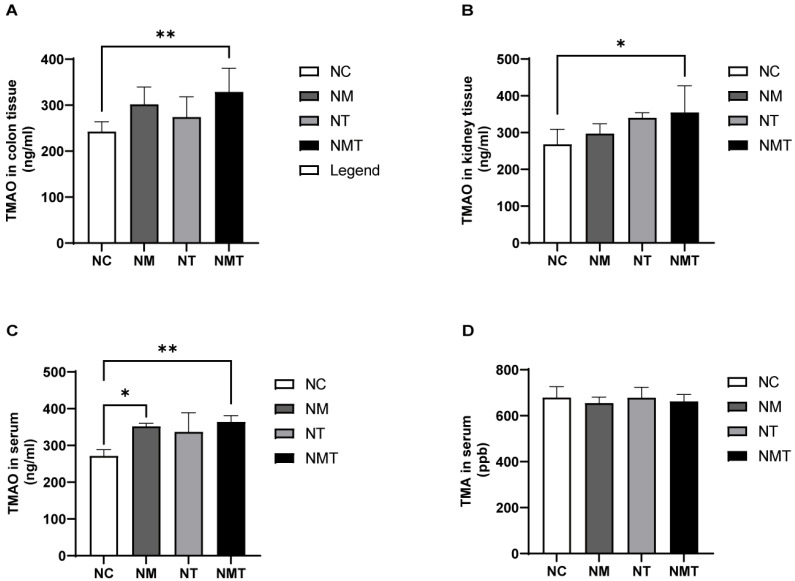
Changes in TMAO and TMA levels in mice across different groups. (**A**) TMAO content in colon tissue; (**B**) TMAO content in kidney tissue; (**C**) TMAO content in serum; (**D**) TMA content in serum. Data are expressed as mean ± SD. * *p* < 0.05; ** *p* < 0.01. NC: normal control group; NM: model group; NT: TMAO group; NMT: TMAO + model group.

As shown in Figure 7A, serum TMAO levels were significantly elevated in the model group compared with the normal control group (*p* < 0.05). Although TMAO levels in colonic and kidney tissues also appeared higher in the model group, these differences did not reach statistical significance (*p* > 0.05) (Figure 7B,C). The TMAO + model group exhibited significantly elevated TMAO levels in colonic tissue and serum (*p* < 0.01), with kidney tissue TMAO levels also showing a notable increase (*p* < 0.05). The TMAO group exhibited an upward trend in TMAO levels across serum, colonic tissue, and kidney tissue (*p* > 0.05). TMA levels in the model group and TMAO + model group were slightly lower than those in the normal control group, but the differences were not statistically significant (*p* > 0.05) (Figure 7D).

### 2.7. Effect of TMAO on Characteristic Microbiota in Mice

**Figure 8:**
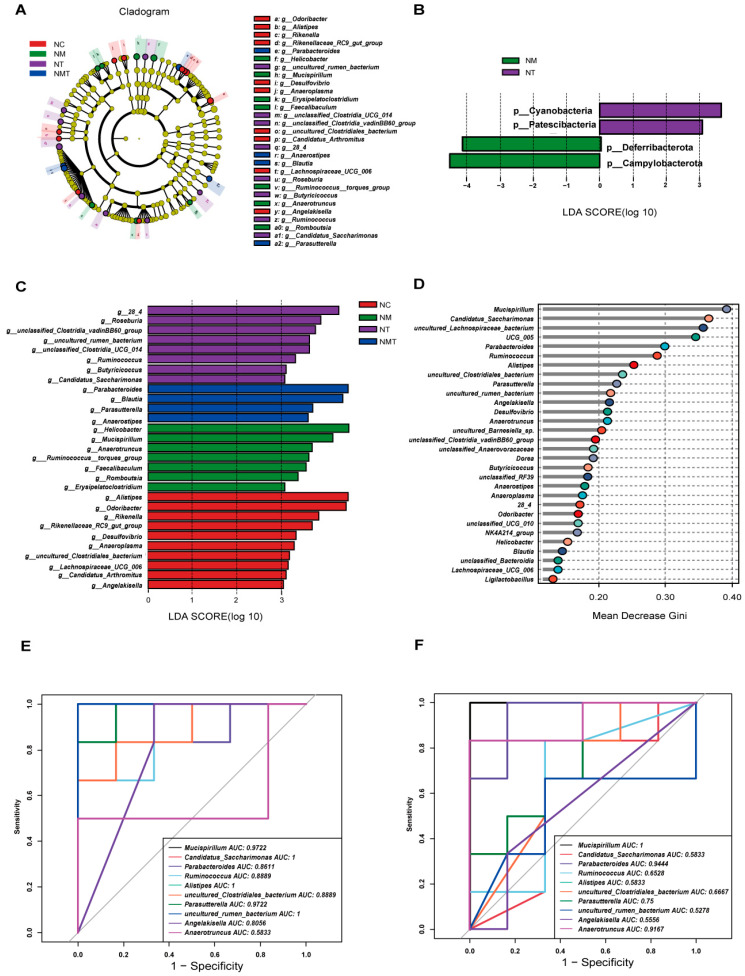
Analysis of intergroup differences in microbial species in cecal contents. (**A**) LDA bar chart at the phylum level. (**B**) Cladogram at the genus level. (**C**) LDA bar chart at the genus level. (**D**) Random forest plot at the genus level. (**E**) ROC curve (NC vs. NM). (**F**) ROC curve (NM vs. NMT) (*n* = 5). NC: normal control group, NM: model group, NT: TMAO group, NMT: TMAO + model group.

LEfSe analysis was conducted to identify differences across all taxonomic levels among groups, with an LDA threshold set at 3. The LDA bar chart illustrates significant differences in phylum-level microbiota abundance in the cecal contents of mice across groups (Figure 8A). *Campylobacterota* and *Deferribacterota* were identified as significantly different phyla in the model group, while *Cyanobacteria* and *Patescibacteria* were significantly different in the TMAO group. The phylogenetic tree (Figure 8B) and the bar plot of genus-level LDA scores (Figure 8C) display species with significant differences in genus-level abundance. In the normal group, significantly different genera included *Alistipes*, *Odoribacter*, and *Rikenella*. The model group demonstrated notable differences in genera including *Helicobacter*, *Mucispirillum*, and *Anaerotruncus*. The TMAO group exhibited seven significantly different genera, including *28_4*, *Roseburia*, and *Ruminococcus*, among others. The TMAO + model group demonstrated significant differences in *Parabacteroides*, *Blautia*, *Parasutterella*, and *Anaerostipes*. Random forest analysis was employed to identify key marker species among groups (Figure 8D). Ten important bacterial genera were selected and ranked by importance from high to low as follows: *Mucispirillum*, *Candidatus_Saccharimonas*, *Parabacteroides*, *Ruminococcus*, *Alistipes*, *uncultured Clostridiales bacterium*, *Parasutterella*, *uncultured rumen bacterium*, *Angelakisella*, and *Anaerotruncus*. ROC analysis was performed on the identified characteristic genera. *Alistipes* (AUC = 1) and *Angelakisella* (AUC = 0.81) displayed high AUC values in the normal group (Figure 8E). In comparisons between the model group and the TMAO + model group, *Mucispirillum* (AUC = 0.97) and *Anaerotruncus* (AUC = 0.92) from the model group, as well as *Parabacteroides* (AUC = 0.86) and *Parasutterella* (AUC = 0.75) from the TMAO + model group, also exhibited high AUC values. Furthermore, characteristic genera of the TMAO group, including *Candidatus_Saccharimonas* (AUC = 1) and *Ruminococcus* (AUC = 0.86), along with the TMAO + model group genera *Parabacteroides* (AUC = 1) and *Parasutterella* (AUC = 0.92), demonstrated high AUC values (Figure 8F).

### 2.8. Correlation Analysis

**Figure 9:**
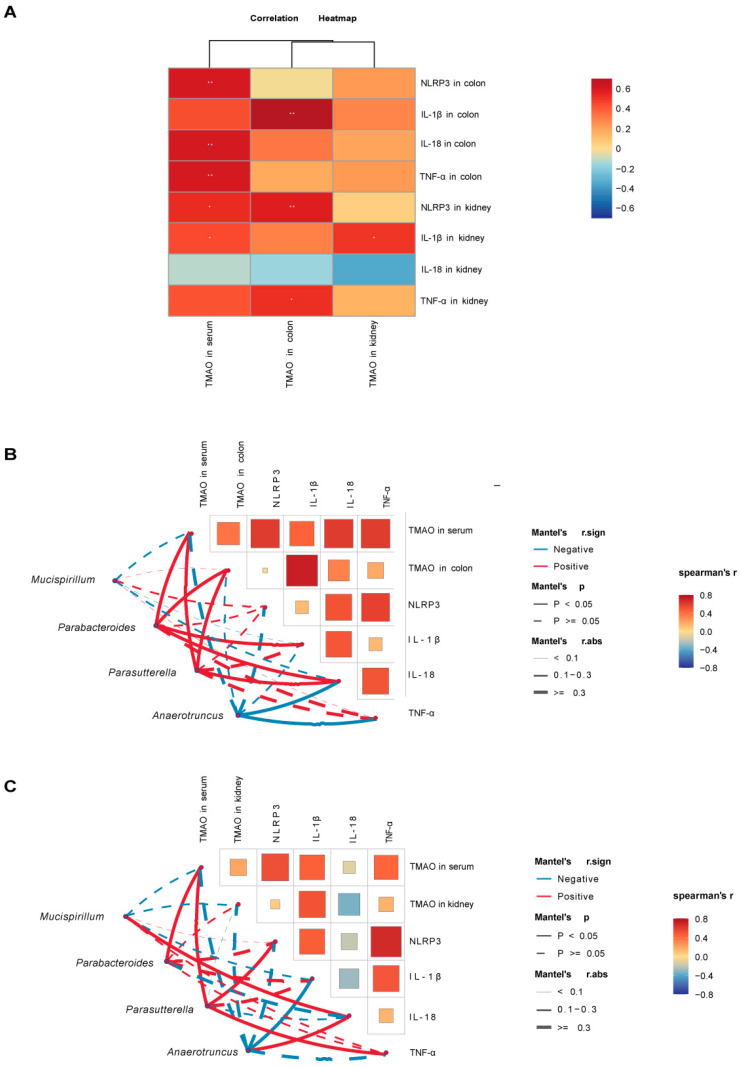
Correlation analysis between TMAO, inflammatory factors, inflammasomes, and characteristic bacterial genera in mice. (**A**) Heatmap showing correlations between TMAO and inflammasomes/inflammatory factors. (**B**) Heatmap showing correlations between colonic TMAO, inflammasomes, inflammatory factors, and characteristic bacterial genera. (**C**) Heatmap showing correlations between kidney TMAO, inflammasomes, inflammatory factors, and characteristic bacterial genera. Red indicates positive correlations, and blue indicates negative correlations, with the gradient reflecting the strength of the correlation. * *p* < 0.05, ** *p* < 0.01. Solid lines denote significant correlations (*p* < 0.05), while dashed lines denote non-significant correlations (*p* > 0.05). Line thickness represents the strength of the correlation. NC: normal group, NM: model group, NT: TMAO group, NMT: TMAO + model group.

We further performed Spearman correlation analysis to investigate the associations between TMAO, inflammasomes, inflammatory factors, and key bacterial genera, aiming to explore the pathological mechanisms through which TMAO influences the gut–kidney axis in diarrhea. The Spearman analysis results revealed a heatmap of the correlations between TMAO and inflammasomes/inflammatory factors (Figure 9A). Serum TMAO showed a highly significant positive correlation with colonic NLRP3, IL-18, and TNF-α (*p* < 0.01) and a significant positive correlation with kidney NLRP3 and IL-1β (*p* < 0.05). Colonic TMAO was highly significantly positively correlated with colonic IL-1β and kidney NLRP3 (*p* < 0.01) and significantly positively correlated with kidney TNF-α (*p* < 0.05). Kidney TMAO showed a significant positive correlation with kidney IL-1β (*p* < 0.05). Serum, colonic, and kidney TMAO demonstrated varying degrees of positive correlations with inflammasomes and inflammatory factors, except for kidney IL-18. In addition, we selected key bacterial genera with high AUC values from the model and TMAO + model groups and analyzed their correlations with TMAO, environmental factors, and inflammatory markers (Figure 9B,C). The characteristic genera *Mucispirillum* and *Anaerotruncus* in the model group were negatively correlated with TMAO, although not statistically significant (*p* > 0.05). *Anaerotruncus* showed a significant negative correlation with colonic IL-18, colonic TNF-α, and kidney IL-1β (*p* < 0.05). In the TMAO + model group, the characteristic genera *Parabacteroides* and *Parasutterella* were significantly positively correlated with serum and colonic TMAO (*p* < 0.05). *Parabacteroides* showed a significant positive correlation with colonic IL-18 and IL-1β (*p* < 0.05), while *Parasutterella* was significantly positively correlated with colonic IL-18, kidney NLRP3, and kidney TNF-α (*p* < 0.05).

## 3. Discussion

### 3.1. TMAO Elevation Is Linked to Gut Microbiota Dysbiosis in Mice with Diarrhea

Foods such as red meat and saltwater fish are abundant in choline, phosphatidylcholine, and L-carnitine [23]. These compounds are metabolized by gut microbiota to produce TMA [24,25]. Hepatic FMO3 converts TMA to TMAO. TMAO is predominantly eliminated by urine, whereas a minor fraction undergoes gradual spontaneous conversion back to TMA [26]. Under normal conditions, TMA levels in the human body are low, but alterations in gut microbiota can directly affect TMA production, leading to changes in TMAO concentrations [27]. Differences in bacterial genera abundance were observed between groups after intervention. The ROC analysis of these genera showed that *Mucispirillum* and *Anaerotruncus* in the model group and *Parabacteroides* and *Parasutterella* in the TMAO + model group had higher AUC values. This suggests that these specific genera may play a key role in the progression of diarrhea. Correlation analysis revealed a negative correlation between *Mucispirillum* and *Anaerotruncus* with TMAO, while *Parabacteroides* and *Parasutterella* exhibited a significant positive correlation with TMAO. Prior research has shown that *Mucispirillum* is involved in TMA metabolism [28]. Probiotics can reduce TMAO levels by modulating the abundance of Lachnospiraceae and Bacteroidaceae at the family level and *Mucispirillum* at the genus level [29]. Similarly, Shirouchi et al. reported that *Anaerotruncus* is negatively correlated with TMAO levels [30]. Patients with ulcerative colitis exhibit an increased abundance of *Parabacteroides*, with changes in TMAO levels [31]. Furthermore, *Parasutterella* has been identified as being associated with TMAO concentrations [32]. These correlations may reflect microbial sensitivity to TMAO or potential roles in its metabolism. Further studies are needed to clarify whether these genera directly respond to or regulate TMAO levels. Thus, we hypothesize that gut microbiota dysbiosis affects microbial metabolism and function, resulting in elevated TMAO levels.

### 3.2. The Aggravation of Diarrhea in Model Mice Is Closely Associated with TMAO Levels

This model showed that colon and kidney tissues were damaged simultaneously in mice with diarrhea [33]. A bidirectional gut–kidney axis has been identified, indicating that compromised intestinal barrier function results in increased permeability, bacterial translocation, and endotoxin release, which aggravates kidney injury. Kidney injury exacerbates intestinal barrier dysfunction, establishing a detrimental cycle that associates intestinal barrier damage with advancing kidney failure [34]. We hypothesize that the gut–kidney interplay may contribute to the persistent and relapsing nature of diarrhea. Patients with diarrhea often exhibit dysbiosis and abnormalities in intestinal metabolites, which are pivotal to the gut–kidney axis [35]. Our previous study indicated increased levels of the intestinal microbial metabolite TMAO in diarrhea mice, suggesting that TMAO might play a role in its pathogenesis [36]. In this study, we observed diarrhea, decreased anal temperature, weight loss, poor mental state, and elevated fecal water content in the model group. The fecal water content in the TMAO + model group was higher than that in the model group. TMAO levels in the model group were elevated compared to the normal group. The TMAO content in the TMAO + model group was the highest among the four groups. The TMA levels in both the model and TMAO + model groups were slightly lower than those in the normal group, probably due to the conversion of most of TMA to TMAO. In addition, elevated TMAO levels may inhibit TMA production. In mice with diarrhea, TMAO levels rose, and diarrhea worsened after TMAO intervention. This shows TMAO’s significant correlation with the development and progression of diarrhea. However, interspecies differences in TMAO metabolism may limit direct translation of these findings to humans, warranting future clinical validation.

### 3.3. TMAO May Contribute to Gut–Kidney Interaction During Diarrhea Progression

In the diarrhea model mice, concurrent injury to both colon and kidney tissues is observed. Histopathological analysis of colonic tissues demonstrated elevated pathological scores, disorganized glandular architecture, diminished goblet cell populations, and varying levels of inflammatory cell infiltration in both the model group and the TMAO + model group. Goblet cells are responsible for mucus secretion, and their depletion results in impaired mucus barrier function [37,38]. ZO-1 and Occludin are key tight junction proteins essential for maintaining intestinal barrier integrity [39]. Furthermore, impaired intestinal barriers release damagesignificant correlation-associated molecules like DAO into the bloodstream, making serum DAO levels a marker of intestinal permeability [39]. This study observed decreased expression of occludin and ZO-1 in the colonic tissues of the model group, alongside increased serum DAO levels, with these changes being more significant in the TMAO + model group. However, to clarify whether these effects are driven directly by TMAO on epithelial cells or mediated indirectly via alterations in the gut microbiota, future studies employing germ-free or antibiotic-treated models, as well as in vitro epithelial cell experiments, are warranted. Elevated TMAO levels correlate with reduced glomerular filtration rate and increased serum creatinine and blood urea nitrogen [40]. The assessment of renal function indicated that BUN and Cr levels were significantly higher in the model group compared to the normal group, with additional exacerbation observed in the TMAO + model group. Histological analysis of the renal tissue indicated inflammatory damage in the kidneys of both the model and TMAO + model groups. The findings indicate that TMAO intervention exacerbates intestinal barrier and renal damage in diarrhea. TMAO has been reported to impair intestinal barrier function through the modulation of inflammasome activity [41]. The NLRP3/Caspase-1 signaling pathway promotes the elevation of inflammatory cytokines such as IL-1β and TNF-α, contributing to oxidative stress and intestinal inflammation [42]. Results of inflammasome (NLRP3, Caspase-1, and Caspase1-p20) and inflammatory cytokines (IL-1β, IL-18, and TNF-α) in colonic and renal tissues revealed significant elevations in these markers within the model group, with even greater increases observed in the TMAO + model group. Correlation analysis demonstrated differing levels of association between TMAO concentrations and NLRP3, along with inflammatory cytokines in colon and kidney tissues. TMAO has been reported to activate the NLRP3 inflammasome and promote the generation of reactive oxygen species (ROS), both of which may exacerbate inflammatory responses [43]. The potential contribution of oxidative stress pathways to gut–kidney injury warrants further investigation. The findings indicate that TMAO may activate inflammasome-related molecules, increase the release of inflammatory cytokines, and contribute to damage in both the intestinal barrier and renal function. Increased intestinal permeability worsens renal damage, whereas progressive renal dysfunction hinders TMAO excretion, resulting in its accumulation and additional intestinal damage. The bidirectional interaction between the gut and kidney may contribute to the ongoing progression of diarrhea. To clarify the underlying causal mechanisms, further studies incorporating specific inhibitors of TMAO production and NLRP3 activation, as well as investigations into downstream molecular pathways, are warranted.

## 4. Materials and Methods

### 4.1. Medication Preparation

Adenine (BioFroxx, Einhausen, Germany, Lot No. EZ65564CEF, HPLC ≥ 99%). Preparation of Adenine Suspension: A suspension was prepared with sterile water at a dosage of 50 mg/(kg·d) and kept fresh for immediate use, away from light. *Folium sennae* (Bozhou Huqiao Pharmaceutical Co., Ltd., Bozhou, China; Lot No. 2312311300022). Preparing the *Folium sennae* decoction: A suitable quantity of *Folium sennae* was weighed and steeped in 10 times the volume of boiling water for 10 min. The mixture was then filtered through gauze and concentrated in a 75 °C water bath to obtain a final crude drug concentration of 1 g/mL. The decoction was kept at 4 °C for further use. Before gavage, the preparation was altered based on the mice’s actual body weight. TMAO (Shanghai Yuanye Biotechnology Co., Ltd., Shanghai, China; Lot No. A06IS222092, HPLC ≥ 98%). Preparing the TMAO Solution: Prepare a sterile solution of water with a dosage of 173 mg/(kg·d) for immediate use.

### 4.2. Grouping and Modeling

Male Kunming SPF mice, aged 6–8 weeks, were obtained from Hunan Silaike Jingda Experimental Animal Co., Ltd., Changsha, China (Animal Quality Certificate No. ZS-202106150014). Male mice were used exclusively to reduce confounding effects related to sex-based differences in gut microbiota [44]. The mice were housed at the Experimental Animal Center of Hunan University of Chinese Medicine (License No. SYXK (Xiang) 2019-0009) under controlled conditions: temperature 23–25 °C, humidity 50–70%, and a 12 h light–dark cycle. The animals had ad libitum access to food and water. The basic diet and the feed ingredients were purchased from Beijing Huafukang Company, Beijing, China (License No. Beijing Feed Certificate 2019-06076). Forty KM mice were randomly divided into four groups: normal group, model group, TMAO group, and TMAO + model group (*n* = 10). 

In this study, we utilized a diarrhea model previously established by our research group. This model is characterized by both intestinal and renal injuries, with pathological mechanisms potentially linked to gut–kidney interactions [20]. possibly involving gut–kidney interaction. The model group mice were administered adenine suspension at a dose of 50 mg/(kg·d) via gavage once daily for 14 consecutive days [45]. Starting from the eighth day, the model group received senna decoction via gavage at a dose of 10 g/(kg·d), once daily for seven days [20]. Mice in the TMAO group and TMAO + model group were given TMAO solution via gavage at a dose of 173 mg/kg·d for 14 days. Mice in the normal group were given an equivalent volume of sterile water via gavage for 14 days. At the end of the trial, the mice were sacrificed via cervical dislocation, and blood, kidney tissues, colon tissues, and cecal contents were taken for investigation of relevant indicators.

### 4.3. General Condition and Fecal Water Content of Mice

The mice’s mental condition, fur appearance (texture and color), and fecal features were observed. The rectal temperature and body weight were measured. The body weight change rate is calculated as (post-experiment weight − pre-experiment weight)/pre-experiment weight × 100%. Fecal samples were collected, weighed, and dried at 105 °C to 110 °C until a consistent weight was reached. The dry weight was recorded. Fecal water content was calculated as (wet weight − dry weight)/wet weight × 100%.

### 4.4. Biochemical Methods

At the end of the modeling process, blood samples were collected from mice using the enucleation method. The samples were left at room temperature for four hours and then centrifuged at 3000 r/min for 15 min at 4 °C to obtain the supernatant. The levels of Cr and BUN were measured using a fully automated biochemical analyzer (BK-280, Shandong Biok Biological Industry Co., Ltd., Zouping, China).

### 4.5. Enzyme-Linked Immunosorbent Assay

Serum samples were used to assess TMAO, TMA, and DAO levels. The colon and kidney tissues were homogenized with a tissue grinder, and then centrifuged at 3000 r/min for 10 min at 4 °C, and the supernatants were collected. The levels of TMAO, NLRP3, caspase-1, caspase-1 p20, IL-1β, IL-18, and tumor necrosis factor-alpha (TNF-α) in the colon and kidney were measured. The sample preparation, enzyme addition, incubation, plate washing, color development, and reaction termination procedures were carried out in accordance with the assay kit manufacturer’s instructions. Optical density values were determined using a microplate reader, and standard curves were created to compute the concentrations of the aforementioned markers in the samples. ELISA kits were procured from Jiangsu Jingmei Biological Technology Co., Ltd., Yancheng, China, NLRP3 (Cat. No. JM-02936M1), caspase-1 (Cat. No. JM-11434M1), caspase-1 p20 (Cat. No. JM-11880M1), IL-1β (Cat. No. JM-02323M1), IL-18 (Cat. No. JM-02452M1), TNF-α (Cat. No. JM-02415M1, Lot NO. 202405), DAO (Cat. No. JM-02511M1), TMAO (Cat. No. JM-11747M1) and TMA (Cat. No. JM-11998M1).

### 4.6. Histological Examination of Colon and Kidney Tissues

Colon and kidney tissue samples collected from mice were fixed in 4% paraformaldehyde solution, dehydrated through a gradient ethanol, cleared in xylene, and embedded in paraffin. Tissue sections were prepared and stained with hematoxylin and eosin (H&E). After staining, the sections were dehydrated again through graded ethanol, cleared in xylene, and mounted with neutral resin. Morphological changes in the colon and kidney tissues were observed under a light microscope.

### 4.7. Immunohistochemical Detection of ZO-1 and Occludin Expression in the Colon

Following dewaxing, the paraffin sections of colonic tissue were immersed in xylene and anhydrous ethanol reagents in succession. The tissue sections were placed in a repair box containing citrate antigen retrieval buffer and microwaved to retrieve the antigen. Sections were then immersed in 3% hydrogen peroxide solution and incubated at room temperature in the dark for 25 min to block endogenous peroxidase activity. After brief drying, a hydrophobic barrier was drawn around the tissue sections using a PAP pen to prevent antibody diffusion. Subsequently, serum blocking was performed. The primary antibodies against ZO-1 (Cat No. AF5145, Affnity, Shanghai, China) and Occludin (Cat No. 27260-1-AP, Proteintech, Rosemont, IL, USA) were added dropwise to the circles and incubated overnight at 4 °C. On the second day, the sections were then incubated with the secondary antibody. To control the staining intensity, the 3,3′-diaminobenzidine (DAB) substrate was applied, and the reaction was observed under a microscope. Once the staining was finished, the nuclei were counterstained with hematoxylin. Finally, the sections were dehydrated and mounted. Positive expression is characterized by a brown or tan coloring. By measuring the cumulative optical density value and the area value of each image, the average optical density (AOD) value was calculated.

### 4.8. Collection of Intestinal Contents and 16S rRNA Sequencing

Since TMA is primarily produced in the cecum, cecal contents were collected for sequencing [46]. Under sterile conditions, the mouse abdominal cavity was opened, and the cecum was removed to obtain cecal content samples. The samples were transferred to 1.5 mL sanitized centrifuge tubes, labeled, weighed, and stored at −80 °C for gut microbiota analysis. Total genomic DNA was extracted from intestinal mucosal samples using a DNA extraction kit (Tiangen Biotech, Beijing, China). The quality and quantity of the extracted DNA were assessed via 1.8% agarose gel electrophoresis, and the concentration and purity were measured using a NanoDrop 2000 UV–Vis spectrophotometer (Thermo Fisher Scientific, Waltham, MA, USA). The hypervariable V3–V4 region of the bacterial 16S rRNA gene was amplified via PCR using forward primer 338F (5′-ACTCCTACGGGAGGCAGCA-3′) and reverse primer 806R (5′-GGACTACHVGGGTWTCTAAT-3′). PCR products were verified on agarose gels and purified using the Omega DNA purification kit (Omega Inc., Norcross, GA, USA). Quantification of purified products was performed using the Qsep-400 system (BiOptic, Inc., New Taipei City, Taiwan, ROC). The amplicon library was sequenced using paired-end sequencing (2 × 250 bp) on the Illumina NovaSeq 6000 platform (Beijing Biomarker Technologies Co., Ltd., Beijing, China).

### 4.9. Characteristic Gut Microbiota Analysis

Linear discriminant analysis (LDA) was performed on the samples under various grouping circumstances. Furthermore, linear discriminant analysis effect size (LEfSe) was used to investigate major taxonomic differences across groups, with discriminative features identified using a logarithmic LDA score threshold of 4.0. Random forest analysis was used to investigate the complicated nonlinear interdependence of variables, resulting in effective, robust, and accurate categorization of microbial community data. The predictive utility of certain gut microbiota was assessed using receiver operating characteristic (ROC) curves and area under the curve (AUC).

### 4.10. Correlation Analysis Method

Spearman correlation coefficients were used to analyze the connections between TMAO and NLRP3, IL-1β, IL-18, and TNF-α. Furthermore, relationships between certain gut flora and TMAO were investigated.

### 4.11. Statistical Analysis

Data processing and statistical analyses were performed using SPSS 27.0 software. The results are shown as mean ± standard deviation (mean ± SD). For data with normality and homogeneity of variance, one-way analysis of variance (ANOVA) was used for group comparisons, followed by post hoc analysis using the LSD method. For data that did not match these criteria, the Kruskal–Wallis H test was used for multiple group comparisons. The significance level was established at α = 0.05, and differences were judged statistically significant with *p* < 0.05.

## 5. Conclusions

**Figure 10:**
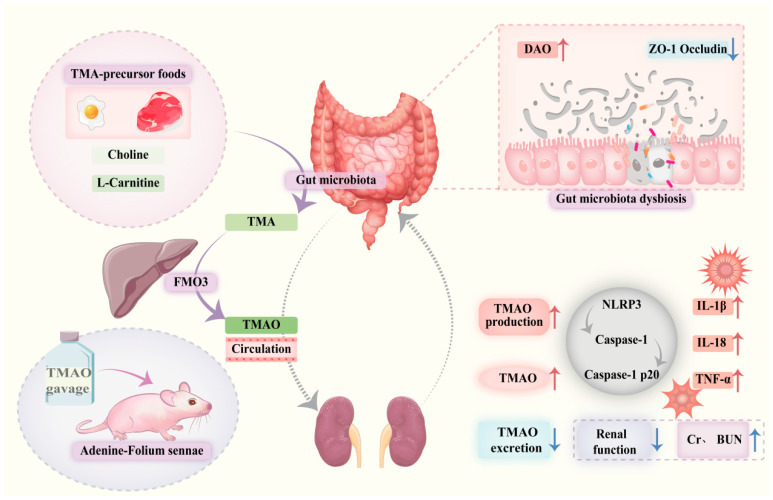
Trimethylamine N-oxide facilitates diarrhea by influencing gut–kidney interaction. Dysbiosis of the gut microbiota leads to reduced expression of ZO-1 and Occludin, resulting in impaired intestinal barrier function and increased DAO levels. Dietary components or other factors (such as modeling interventions) increase TMAO production and elevate systemic TMAO levels.. Elevated TMAO activates the NLRP3 inflammasome, promoting release of inflammatory cytokines including IL-1β, IL-18, and TNF-α, which aggravate renal inflammation and cause elevated Cr and BUN levels. Impaired kidney function reduces TMAO excretion, creating a positive feedback loop that further raises systemic TMAO, exacerbating intestinal and renal injury and contributing to diarrhea pathogenesis.

In summary, TMAO can activate inflammasomes, promote the release of inflammatory cytokines, and induce inflammatory damage in the colon and kidney tissues. TMAO contributes to impaired intestinal barrier function and renal dysfunction, playing a role in the gut–kidney interaction observed in the diarrhea mouse model. The gut microbiota-TMAO-inflammasome-inflammatory cytokine axis might serve as a crucial mechanism underlying the development and progression of intestinal inflammation (Figure 10). This study highlights the impact of TMAO on gut–kidney interactions, providing novel insights into the pathogenesis of intestinal diseases. Moreover, it establishes an experimental basis for further exploring the role of gut microbiota-derived metabolites in the development and progression of gut–organ axis-related disorders.

## Data Availability

The gut microbiome sequencing data has been uploaded to the NCBI database (https://www.ncbi.nlm.nih.gov/, accessed on 22 December 2024), no. PRJNA1201605.

## Abbreviations

The following abbreviations are used in this manuscript:

TMAO	trimethylamine N-oxide
CKD	chronic kidney disease
TMA	trimethylamine
FMO3	flavin-containing monooxygenase 3
ZO-1	zonula occludens-1
DAO	diamine oxidase
Cr	creatinine
BUN	blood urea nitrogen
NLRP3	nucleotide-binding oligomerization domain-like receptor protein
IL-1β	interleukin-1 beta
IL-18	interleukin-18
TNF-α	tumor necrosis factor-alpha
H&E	hematoxylin and eosin
DAB	3,3′-diaminobenzidine
AOD	average optical density
LDA	linear discriminant analysis
ROC	receiver operating characteristic
AUC	area under the curve

## References

[1] Xiao L., Liu Q., Luo M., Xiong L. (2021). Gut Microbiota-Derived Metabolites in Irritable Bowel Syndrome. Front. Cell. Infect. Microbiol..

[2] Gong J., Noel S., Pluznick J.L., Hamad A.R.A., Rabb H. (2019). Gut Microbiota-Kidney Cross-Talk in Acute Kidney Injury. Semin. Nephrol..

[3] Chen Y.Y., Chen D.Q., Chen L., Liu J.R., Vaziri N.D., Guo Y., Zhao Y.Y. (2019). Microbiome-metabolome reveals the contribution of gut-kidney axis on kidney disease. J. Transl. Med..

[4] Rysz J., Franczyk B., Ławiński J., Olszewski R., Ciałkowska-Rysz A., Gluba-Brzózka A. (2021). The Impact of CKD on Uremic Toxins and Gut Microbiota. Toxins.

[5] Kalantar-Zadeh K., Lockwood M.B., Rhee C.M., Tantisattamo E., Andreoli S., Balducci A., Laffin P., Harris T., Knight R., Kumaraswami L. (2022). Patient-centred approaches for the management of unpleasant symptoms in kidney disease. Nat. Rev. Nephrol..

[6] Belo L., Carvalho M. (2023). Chronic Kidney Disease: Underlying Molecular Mechanisms-A Special Issue Overview. Int. J. Mol. Sci..

[7] Shin H.S., Chandraker A. (2017). Causes and management of postrenal transplant diarrhea: An underappreciated cause of transplant-associated morbidity. Curr. Opin. Nephrol. Hypertens..

[8] Gioco R., Corona D., Ekser B., Puzzo L., Inserra G., Pinto F., Schipa C., Privitera F., Veroux P., Veroux M. (2020). Gastrointestinal complications after kidney transplantation. World J. Gastroenterol..

[9] Gatarek P., Kaluzna-Czaplinska J. (2021). Trimethylamine N-oxide (TMAO) in human health. EXCLI J..

[10] Banno Y., Nomura M., Hara R., Asami M., Tanaka K., Mukai Y., Tomata Y. (2023). Trimethylamine N-oxide and risk of inflammatory bowel disease: A Mendelian randomization study. Medicine.

[11] Rath S., Rud T., Pieper D.H., Vital M. (2019). Potential TMA-Producing Bacteria Are Ubiquitously Found in Mammalia. Front. Microbiol..

[12] Chen Y., Weng Z., Liu Q., Shao W., Guo W., Chen C., Jiao L., Wang Q., Lu Q., Sun H. (2019). FMO3 and its metabolite TMAO contribute to the formation of gallstones. Biochim. Biophys. Acta Mol. Basis Dis..

[13] Xie J., Ma X., Zheng Y., Mao N., Ren S., Fan J. (2022). Panax notoginseng saponins alleviate damage to the intestinal barrier and regulate levels of intestinal microbes in a rat model of chronic kidney disease. Ren. Fail..

[14] Yu P.S., Wu P.H., Hung W.W., Lin M.Y., Zhen Y.Y., Hung W.C., Chang J.M., Tsai J.R., Chiu Y.W., Hwang S.J. (2024). Association Between Trimethylamine N-oxide and Adverse Kidney Outcomes and Overall Mortality in Type 2 Diabetes Mellitus. J. Clin. Endocrinol. Metab..

[15] Lai Y., Tang H., Zhang X., Zhou Z., Zhou M., Hu Z., Zhu F., Zhang L., Nie J. (2022). Trimethylamine-N-Oxide Aggravates Kidney Injury via Activation of p38/MAPK Signaling and Upregulation of HuR. Kidney Blood Press. Res..

[16] Yong C., Huang G., Ge H., Zhu Y., Yang Y., Yu Y., Tian F., Gao K., Zhou E. (2023). Perilla frutescens L. alleviates trimethylamine N-oxide-induced apoptosis in the renal tubule by regulating ASK1-JNK phosphorylation. Phytother. Res..

[17] Ge H., Wei Y., Zhang W., Yong C., Chen Y., Zhou E. (2024). Suyin Detoxification Granule alleviates trimethylamine N-oxide-induced tubular ferroptosis and renal fibrosis to prevent chronic kidney disease progression. Phytomedicine.

[18] Rodrigues C., Ismael S., Castela I., Barreiros-Mota I., Almeida M.J., Santos G.M., Calhau C., Rocha J.C., Faria A., Araújo J.R. (2023). Trimethylamine increases intestinal fatty acid absorption: In vitro studies in a Caco-2 cell culture system. J. Nutr. Sci..

[19] Xie S., Fang L., Deng N., Shen J., Tan Z., Peng X. (2024). Targeting the Gut-Kidney Axis in Diarrhea with Kidney-Yang Deficiency Syndrome: The Role of Sishen Pills in Regulating TMAO-Mediated Inflammatory Response. Med. Sci. Monit..

[20] Li X., Peng X., Qiao B., Peng M., Deng N., Yu R., Tan Z. (2022). Gut-Kidney Impairment Process of Adenine Combined with Folium sennae-Induced Diarrhea: Association with Interactions between Lactobacillus intestinalis, Bacteroides acidifaciens and Acetic Acid, Inflammation, and Kidney Function. Cells.

[21] Liu Y., Ye Q., Liu Y.L., Kang J., Chen Y., Dong W.G. (2017). Schistosoma japonicum attenuates dextran sodium sulfate-induced colitis in mice via reduction of endoplasmic reticulum stress. World J. Gastroenterol..

[22] Wang Y., Zhang J., Zhang B., Lu M., Ma J., Liu Z., Huang J., Ma J., Yang X., Wang F. (2023). Modified Gegen Qinlian decoction ameliorated ulcerative colitis by attenuating inflammation and oxidative stress and enhancing intestinal barrier function in vivo and in vitro. J. Ethnopharmacol..

[23] Wang Z., Bergeron N., Levison B.S., Li X.S., Chiu S., Jia X., Koeth R.A., Li L., Wu Y., Tang W.H.W. (2019). Impact of chronic dietary red meat, white meat, or non-meat protein on trimethylamine N-oxide metabolism and renal excretion in healthy men and women. Eur. Heart J..

[24] Li J., Li Y., Ivey K.L., Wang D.D., Wilkinson J.E., Franke A., Lee K.H., Chan A., Huttenhower C., Hu F.B. (2022). Interplay between diet and gut microbiome, and circulating concentrations of trimethylamine N-oxide: Findings from a longitudinal cohort of US men. Gut.

[25] Cho C.E., Caudill M.A. (2017). Trimethylamine-N-Oxide: Friend, Foe, or Simply Caught in the Cross-Fire?. Trends Endocrinol. Metab..

[26] Jia X., Osborn L.J., Wang Z. (2020). Simultaneous Measurement of Urinary Trimethylamine (TMA) and Trimethylamine N-Oxide (TMAO) by Liquid Chromatography-Mass Spectrometry. Molecules.

[27] Yu Y., Yin Y., Deng J., Yang X., Bai S., Yu R. (2024). Unveiling the causal effects of gut microbiome on trimethylamine N-oxide: Evidence from Mendelian randomization. Front. Microbiol..

[28] Li X., Song J., Shi X., Huang M., Liu L., Yi G., Yang N., Xu G., Zheng J. (2022). FMO3 deficiency of duck leads to decreased lipid deposition and increased antibacterial activity. J. Anim. Sci. Biotechnol..

[29] Qiu L., Tao X., Xiong H., Yu J., Wei H. (2018). Lactobacillus plantarum ZDY04 exhibits a strain-specific property of lowering TMAO via the modulation of gut microbiota in mice. Food Funct..

[30] Shirouchi B., Fukuda A., Akasaka T. (2022). Unlike Glycerophosphocholine or Choline Chloride, Dietary Phosphatidylcholine Does Not Increase Plasma Trimethylamine-N-Oxide Levels in Sprague-Dawley Rats. Metabolites.

[31] Laryushina Y., Samoilova-Bedych N., Turgunova L., Kozhakhmetov S., Alina A., Suieubayev M., Mukhanbetzhanov N. (2024). Alterations of the Gut Microbiome and TMAO Levels in Patients with Ulcerative Colitis. J. Clin. Med..

[32] Zysset-Burri D.C., Keller I., Berger L.E., Neyer P.J., Steuer C., Wolf S., Zinkernagel M.S. (2019). Retinal artery occlusion is associated with compositional and functional shifts in the gut microbiome and altered trimethylamine-N-oxide levels. Sci. Rep..

[33] Li X., Qiao B., Wu Y., Deng N., Yuan J., Tan Z. (2024). Sishen Pill inhibits intestinal inflammation in diarrhea mice via regulating kidney-intestinal bacteria-metabolic pathway. Front. Pharmacol..

[34] Pahl M.V., Vaziri N.D. (2015). The Chronic Kidney Disease–Colonic Axis. Semin. Dial..

[35] Huang Y., Xin W., Xiong J., Yao M., Zhang B., Zhao J. (2022). The Intestinal Microbiota and Metabolites in the Gut-Kidney-Heart Axis of Chronic Kidney Disease. Front. Pharmacol..

[36] Guo M., Wu Y., Peng M., Xiao N., Lei Z., Tan Z. (2024). Decreasing of Trimethylamine N-Oxide by Cecal Microbiota and Choline-Trimethylamine Lyase are Associated with Sishen Pill on Diarrhea with Kidney-Yang Deficiency Syndrome. J. Inflamm. Res..

[37] Gustafsson J.K., Johansson M.E.V. (2022). The role of goblet cells and mucus in intestinal homeostasis. Nat. Rev. Gastroenterol. Hepatol..

[38] Nian F., Chen Y., Xia Q., Zhu C., Wu L., Lu X. (2024). Gut microbiota metabolite trimethylamine N-oxide promoted NAFLD progression by exacerbating intestinal barrier disruption and intrahepatic cellular imbalance. Int. Immunopharmacol..

[39] Linsalata M., Riezzo G., Clemente C., D’Attoma B., Russo F. (2020). Noninvasive Biomarkers of Gut Barrier Function in Patients Suffering from Diarrhea Predominant-IBS: An Update. Dis. Markers.

[40] Zeng Y., Guo M., Fang X., Teng F., Tan X., Li X., Wang M., Long Y., Xu Y. (2021). Gut Microbiota-Derived Trimethylamine N-Oxide and Kidney Function: A Systematic Review and Meta-Analysis. Adv. Nutr..

[41] Yue C., Yang X., Li J., Chen X., Zhao X., Chen Y., Wen Y. (2017). Trimethylamine N-oxide prime NLRP3 inflammasome via inhibiting ATG16L1-induced autophagy in colonic epithelial cells. Biochem. Biophys. Res. Commun..

[42] Chen X., Kong Q., Zhao X., Zhao C., Hao P., Irshad I., Lei H., Kulyar M.F., Bhutta Z.A., Ashfaq H. (2022). Sodium acetate/sodium butyrate alleviates lipopolysaccharide-induced diarrhea in mice via regulating the gut microbiota, inflammatory cytokines, antioxidant levels, and NLRP3/Caspase-1 signaling. Front. Microbiol..

[43] Chen M.L., Zhu X.H., Ran L., Lang H.D., Yi L., Mi M.T. (2017). Trimethylamine-N-Oxide Induces Vascular Inflammation by Activating the NLRP3 Inflammasome Through the SIRT3-SOD2-mtROS Signaling Pathway. J. Am. Heart Assoc..

[44] Wu Y., Peng X., Li X., Li D., Tan Z., Yu R. (2022). Sex hormones influence the intestinal microbiota composition in mice. Front. Microbiol..

[45] Fang L., Shen J., Wu Y., Tan Z. (2025). Involvement of intestinal mucosal microbiota in adenine-induced liver function injury. 3 Biotech.

[46] Koeth R.A., Levison B.S., Culley M.K., Buffa J.A., Wang Z., Gregory J.C., Org E., Wu Y., Li L., Smith J.D. (2014). γ-Butyrobetaine is a proatherogenic intermediate in gut microbial metabolism of L-carnitine to TMAO. Cell Metab..

